# NRK-ABMIL: Subtle Metastatic Deposits Detection for Predicting Lymph Node Metastasis in Breast Cancer Whole-Slide Images

**DOI:** 10.3390/cancers15133428

**Published:** 2023-06-30

**Authors:** Usama Sajjad, Mostafa Rezapour, Ziyu Su, Gary H. Tozbikian, Metin N. Gurcan, M. Khalid Khan Niazi

**Affiliations:** 1 Center for Biomedical Informatics, Wake Forest University School of Medicine, Winston-Salem, NC 27101, USA; mrezapou@wakehealth.edu (M.R.); zsu@wakehealth.edu (Z.S.); mgurcan@wakehealth.edu (M.N.G.); mniazi@wakehealth.edu (M.K.K.N.); 2 Department of Pathology, The Ohio State University, Columbus, OH 43210, USA; gary.tozbikian@osumc.edu

**Keywords:** deep learning, histopathology, multiple-instance learning, breast cancer

## Abstract

**Simple Summary:**

Recent advancements in AI have revolutionized cancer research, especially in the analysis of histopathological imaging data with minimal human involvement. Early detection of lymph node metastasis in breast cancer is vital for treatment outcomes. This paper introduces a novel approach that combines representation learning and deep learning (DL) to detect small tumors (STs) without neglecting larger ones. The proposed method uses representation learning to identify STs in histopathology images, followed by DL algorithms for breast cancer detection. Extensive evaluation shows remarkable accuracy in detecting STs without compromising larger-lesion detection. This approach enables early detection, timely intervention, and potentially improved treatment outcomes. The integration of representation learning and DL offers a promising solution for ST detection in breast cancer. By reducing human involvement and leveraging AI capabilities, the proposed method achieves impressive accuracy in identifying STs. Further research and validation could enhance diagnostic capabilities and personalized treatment strategies, ultimately benefiting breast cancer patients.

**Abstract:**

The early diagnosis of lymph node metastasis in breast cancer is essential for enhancing treatment outcomes and overall prognosis. Unfortunately, pathologists often fail to identify small or subtle metastatic deposits, leading them to rely on cytokeratin stains for improved detection, although this approach is not without its flaws. To address the need for early detection, multiple-instance learning (MIL) has emerged as the preferred deep learning method for automatic tumor detection on whole slide images (WSIs). However, existing methods often fail to identify some small lesions due to insufficient attention to small regions. Attention-based multiple-instance learning (ABMIL)-based methods can be particularly problematic because they may focus too much on normal regions, leaving insufficient attention for small-tumor lesions. In this paper, we propose a new ABMIL-based model called normal representative keyset ABMIL (NRK-ABMIL), which addresseses this issue by adjusting the attention mechanism to give more attention to lesions. To accomplish this, the NRK-ABMIL creates an optimal keyset of normal patch embeddings called the normal representative keyset (NRK). The NRK roughly represents the underlying distribution of all normal patch embeddings and is used to modify the attention mechanism of the ABMIL. We evaluated NRK-ABMIL on the publicly available Camelyon16 and Camelyon17 datasets and found that it outperformed existing state-of-the-art methods in accurately identifying small tumor lesions that may spread over a few patches. Additionally, the NRK-ABMIL also performed exceptionally well in identifying medium/large tumor lesions.

## 1. Introduction

Histopathological tissue analysis is a crucial tool for diagnosing various diseases [1]. With the increasing use of digital whole slide image (WSI) scanners, histopathology analysis has transitioned from glass slides to digital images, which has made the analysis process more convenient [2,3]. WSIs typically have extremely high resolutions, allowing pathologists to analyze tissues at high magnification. However, due to the huge size of WSIs, manual diagnosis and prognosis can be a tedious and time-consuming process, which has sparked interest in exploring deep learning-based methods in digital pathology [4,5,6]. Despite the potential advantages of deep learning-based methods, conventional, fully supervised deep learning methods face several challenges when applied to histopathology analysis. For instance, the gigapixel resolution of WSIs and the inaccessibility of pixel-level annotations, which are diagnostic labels annotated by pathologists, pose significant challenges [7]. Due to the presence of inter-reader variability among pathologists, it can be challenging to define the lesions in a way that is suitable for fully supervised learning methods.

To address these challenges, recent algorithms [8,9] have employed the multiple-instance learning (MIL) paradigm to analyze WSIs [10]. In MIL, the input of the model is a collection of data instances, referred to as a “bag”, and the output is the prediction of the bag. Unlike fully supervised learning methods, weak labels are assigned to the bag rather than the individual instances [11]. In the MIL formulation, WSIs are divided into small, often non-overlapping patches, which are analyzed separately by neural networks. The aggregated results of the small patches are used to perform slide-level classification. Using MIL has proven to be a promising approach for histopathology analysis, enabling the identification of important features for classification and alleviating the need for extensive manual annotation. By breaking down the analysis of WSIs into small patches, MIL-based methods can achieve accurate and efficient classification without relying on fully supervised learning methods. As such, MIL-based approaches have the potential to significantly improve the speed and accuracy of histopathology analysis, ultimately leading to better disease diagnosis and treatment [12,13].

Current methods for MIL in analyzing WSIs assume that all patches within a WSI are equally important for slide-level prediction. These methods compute attention weights for each patch and use weighted combinations of patch features to derive a meta-representation of the WSI [8,9,10,14]. However, for cases with small lesions, the slide-level label may correspond to only a few patches, making it difficult for existing approaches to identify those important patches. Some methods attempt to train a patch-level classifier to identify these regions and feed them into deep learning models [15,16,17], but this approach is not effective when slide-level labels correspond to only a few patches.

**Figure 1:**
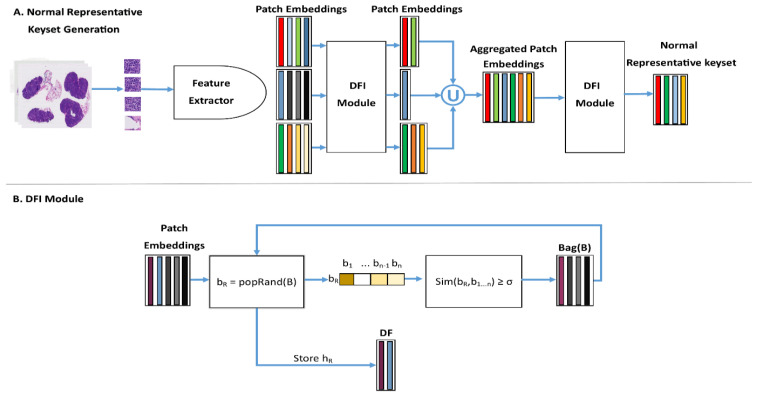
The schematic diagram for constructing NRK. (**A**) NRKG: The input is all normal WSIs and the output, distinct features (DF), is the set of all distinct normal patch embeddings extracted using it. (**B**) The distinct features identifier (DFI) module. popRand(.) is a function that randomly selects one element of its input set, i.e., b_r_, and stores b_r_ as the distinct embedding. Sim(.) is a function that computes the similarity of b_r_, with b_n−1_ embeddings, and removes the embeddings with similarity greater than σ from the bag.

**Figure 2:**
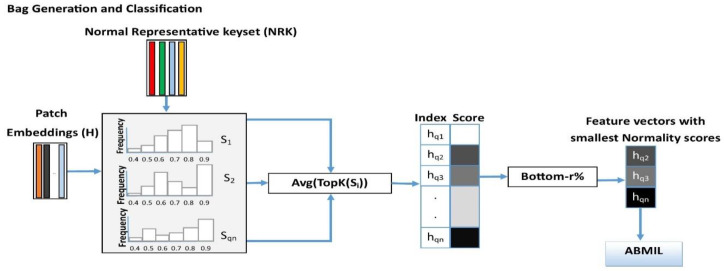
Bag generation and classification. We compare the WSI patch embeddings with NRK, compute the average of TopK similarity scores to compute the normality score of each patch embedding, and select bottom-r% embeddings of a WSI as the input of the ABMIL model.

To address this issue, we propose a new MIL model and demonstrate its effectiveness through the problem of breast cancer metastasis classification in the lymph nodes (BCLNM). The key idea of the proposed method is the use of normal patches that are part of normal WSIs to learn a keyset of representative normal patches. We then design a keyset-based approach that can guide the MIL model to select discriminative patches from WSIs intelligently. The systematic overview of the normal representative keyset generation module (NRKG) is presented in Figure 1. Figure 2 demonstrates the intelligent selection of uncertain feature embeddings for a WSI-level label prediction.

The rest of the manuscript is organized as follows. We discuss related work in Section 2. This is followed by the introduction of the proposed normal representative keyset ABMIL (NRK-ABMIL) model. We present the results in Section 4, and discuss them in Section 5. The proposed method offers a promising solution to the challenge of identifying important patches in WSIs with small lesions, and we believe it has the potential to improve the accuracy of breast cancer metastasis classification.

## 2. Related Work

Several machine learning methods that use multiple-instance learning (MIL) techniques employ an attention mechanism for aggregating patch embeddings [8,10,17]. One such method is the attention-based ML (ABMIL) proposed by Ilse et al. [5] for classifying whole slide images (WSI). This method learns to weight the patch embeddings based on their importance in predicting slide-level outcomes. Another method, proposed by Lu et al. [8], incorporates a clustering-based constraint to the ABMIL. This approach uses multiple attention branches to refine the feature space and improve convergence. Shao et al. [9] introduced TransMIL, a method that explores the morphological and spatial relationships between instances for classification using the Transformer architecture [18]. The Transformer architecture is widely used in natural language processing, but it has also shown promise in image-based tasks such as object detection and segmentation [10]. In TransMIL, the Transformer is used to capture the contextual relationship between patches within a slide to improve the accuracy of slide-level predictions.

Our experiments (see Section 4: Results) have revealed that the aforementioned ABMIL-based methods are unable to detect and identify small lesions accurately, for instance, in lymph node metastasis from breast cancer. To overcome this challenge, several MIL methods have been proposed to predict slide-level outcomes based on a few important patches (tumor patches from lymph nodes). For example, Courtiol et al. [16] proposed selecting patches with the highest and lowest scores for slide-level prediction in an end-to-end manner. Campanella et al. [19] stacked the patch identification model and the MIL model into the same stream to select high-probability patches for MIL classification based on a recurrent neural network aggregation function. Li et al. [14] proposed a dual-stream attention mechanism to jointly learn patch classifier and slide classifier and select “critical instance” from each WSI for classification. However, these methods may not be effective in identifying small lesions because slide-level labels are not informative enough to guide models to select suspicious tumor patches from small lesions, which is known as the noisy training problem [8].

In one of our previous works, we proposed attention2majority [17], which trains the discriminator to intelligently sample the patches from lesion regions to overcome the noisy training issue. However, this method requires training the discriminator with WSIs whose slide-level labels correspond to the majority of the tissue area [17]. For instance, the training of this method necessitates whole slide images of tumors where the tumor comprises the majority of the tissue.

These approaches highlight the challenges of identifying small lesions in MIL-based WSI classification and the importance of addressing the noisy training problem. They also demonstrate the potential of unsupervised learning and representation learning to improve the selection of informative patches for MIL models. In this work, we address the limitations of these methods and develop more effective strategies for identifying and classifying small lesions in WSIs.

## 3. Materials and Methods

This section presents a novel attention-based MIL method that uses patch-level labels from normal WSIs to improve the accuracy of WSI-level label classification. We first introduce the dataset used in our experiments and some detail of the clinical problem that we are aiming to solve. We then provide a brief overview of MIL and attention-based MIL (ABMIL) methods for WSI-level label classification. Next, we describe how we leverage known patch-level labels of normal WSIs to create an accurate representative bag for all normal WSI patches, which we refer to as the normal representative keyset (NRK). We explain how we use the NRK to enhance the classification of WSI-level labels. Finally, we discuss how the proposed method identifies and separates patches with high similarity scores to the NRK when given a WSI at inference time. The proposed method utilizes known patch-level labels from normal WSIs to create a representative bag of normal WSI patches. This allows for improved classification of WSI-level labels, particularly in cases where small lesions may be present. We discuss the specific details of the approach, including how we leverage the NRK to enhance classification accuracy and how we effectively identify and separate patches with high similarity scores to the NRK during inference.

### 3.1. Dataset

We evaluate the efficiency of the proposed method on publicly available WSI datasets of lymph node metastasis from breast cancer, namely, Camelyon16 [20] and Camelyon17 [21]. Lymph node metastasis from breast cancer is significant because it is an indication that the cancer cells have spread beyond the breast tissue and into the lymphatic system, which is a network of vessels and organs that help the body fight infection and disease. Lymph nodes are small, bean-shaped structures that filter lymph fluid and are an important part of the immune system. The presence of cancer cells in the lymph nodes means that cancer has the potential to spread further to other parts of the body through the bloodstream. The number of lymph nodes involved and the extent of lymph node involvement can help determine the stage of breast cancer and guide treatment decisions [22]. Camelyon16 consists of a training set of 270 WSIs and an official hold-out test set of 129 WSIs that are sampled from 399 patients [20]. Camelyon17 consists of a training set of 500 WSIs and a hold-out set of 500 WSIs [21] collected from 200 patients. To prepare the dataset for our analysis, we first apply color thresholding to extract the tissue region of the WSI [23]. We then extract non-overlapping patches of size 224 × 224 on 20× magnification.

### 3.2. MIL Method for WSI Classification

We now describe how the MIL method [10] learns to differentiate between normal (negative) and tumor (positive) WSIs (bags). Suppose the training set contains P gigapixel-sized WSIs (bags), $X=\{X_{1},X_{2},\ldots ,X_{P}\}$, with known labels $Y={\{Y}_{1},Y_{2},\ldots ,Y_{P}\}$, where $Y_{i}\in \{0,1\}$ for i=1,…,P, and 0, 1 corresponds to the labels of normal, and tumor bags, respectively. Since WSIs are too large to fit on a GPU, MIL methods tile WSI $X_{i}$, for i=1,…,P, into computationally friendly patches (instances) $X_{i}={\{x}_{i1},x_{i2},\ldots ,x_{in_{i}}\}$, where $n_{i}$ is the number of patches (instances) within the $i^{th}$ WSI [24]. If $y_{ij}$ ∈{0,1} denotes a patch-level label of $x_{ij}\in X_{i}$, for $j=1,\ldots ,n_{i}$, then the WSI-level label of the $i^{th}$ WSI can be formulated as:

(1) $$Y_{i}=\left\{\begin{array}{l} 0,\;if\sum_{j}y_{ij}=0\\ 1\;otherwise \end{array}\right.$$

However, for a tumor WSI (positive bag) $X_{t}$, the patch-level labels $y_{tj}$, for all $j=1,\ldots ,n_{t}$, are unknown. ABMIL method often predict WSI-level labels by

(2) $${\tilde{Y}}_{i}=g\left(\sigma \left(f\left(x_{i1}\right),\ldots ,f\left(x_{in_{i}}\right)\right)\right)$$

where ${\tilde{Y}}_{i}$ is a predicted WSI-level label of the $i^{th}$ WSI, f(·) is a patch-level embedding encoder, σ(·) is an aggregation function, and g(·) is a bag-level prediction classifier. Minimizing a loss function, e.g., the cross entropy, MIL methods finally search for optimal parameters of the classifier g.

### 3.3. Attention-Based MIL (ABMIL) Method for WSI Classification

Following the MIL paradigm, the attention-based MIL method [10] first utilizes a multilayer neural network as a patch-level embedding encoder that transforms each patch $x_{ij}\in X_{i}$ into a patch-level embedding $h_{ij}\in R^{D}$. Then, an attention-based aggregation function is employed to produce a WSI-level embedding $z_{i}$,

(3) $$z_{i}=\sigma \left(h_{i1},h_{i2},\ldots ,h_{in_{i}}\right)=\sum_{j=1}^{n_{i}}a_{ij}h_{ij}\in R^{D}$$

where

(4) $$a_{ij}=\frac{exp\left(W^{T}\left(tanh\left(V^{T}h_{ij}\right)⊙sigm\left(U^{T}h_{ij}\right)\right)\right)}{\sum_{k=1}^{n_{i}}exp\left(W^{T}\left(tanh\left(V^{T}h_{ik}\right)⊙sigm\left(U^{T}h_{ik}\right)\right)\right)}\in R$$

is the attention score corresponding to the patch $x_{ij}$, $V\in R^{D\times L}$, $U\in R^{D\times L}$, $W\in R^{L\times 1}$ are the learnable weights of fully connected networks, where L is the number of neurons in the hidden layer, and ⊙ representsanelement−wisemultiplication. Finally, another fully connected layer neural network, g(⋅), with sigmoid function as the last layer activation function, is employed as a classifier to map $z_{i}$ to a WSI-level class label ${\tilde{Y}}_{i}$.

### 3.4. Normal Representative Keyset (NRK)

Since attention scores obtained via Equation (4) are always nonzero, ABMIL methods (even well-performing ones) assign positive attention scores to normal patches within a tumor WSI. For medium and large tumor WSIs (WSIs with medium and large lesions), assigning positive attention scores to normal patches may not affect the overall ABMIL-based WSI-level label prediction because there is a relatively proper balance between the numbers of normal and tumor patch-level embeddings. However, when it comes to small tumor WSIs (WSIs with small lesions), positive attention scores to normal patches can lessen the impacts of a few tumor-patch-level embeddings in the WSI-level embedding given in Equation (3). As a result, the WSI-level embedding of a small tumor WSI becomes similar to a WSI-level embedding of a normal WSI. Therefore, fewer tumor patches (smaller lesions) within a tumor WSI raise the likelihood of a false-negative decision.

To maintain adequate attention to tumor-patch-level embeddings within a tumor WSI, and ensure that they have a strong effect on the WSI-level embedding given in Equation (3), we need to assign a zero-attention score to normal-patch-level embeddings. Due to SoftMax function properties and derived attention scores in Equation (4), we must identify normal patches within tumor WSIs and remove them before SoftMax function is applied to them. However, this is not directly possible because of the lack of patch-level annotation within tumor WSIs. One way to identify normal-patch-level embeddings within a tumor WSI is to roughly learn their underlying distribution using all normal patches cropped from all normal WSIs. Note that we leverage known patch-level labels of normal WSIs to construct an optimal normal representative keyset.

We now introduce a novel method for constructing the normal representative keyset (NRK) using an NRKG module that consists of distinct normal-patch-level embeddings. In other words, via a controlled cosine similarity-based contrastive process among normal-patch-level embeddings of all normal WSIs, the NRK is constructed to be the smallest distinct set representing the normal patch-level embeddings containing all distinct normal-patch-level embeddings. Note that the NRK construction process is offline, and hence it does not add any online computational cost. Without loss of generality, suppose there are N normal WSIs and T=P−N tumor WSIs in the training set. For the sake of simplicity, suppose $X={\{X}_{1},X_{2},\ldots ,X_{N},X_{N+1},\ldots ,X_{P}\}$ is sorted in a way that the first N WSIs, $X^{Normal}={\{X}_{1},X_{2},\ldots ,X_{N}\}\subset X$, are the subset containing all normal WSIs in the training set. Moreover, let $X_{i}={\{x}_{i1},x_{i2},\ldots ,x_{in_{i}}\}$ and $H_{i}=${$h_{i1},h_{i2},.h_{in_{i}}$}, for i=1,…,N, be the set of patches and patch-level embeddings of the $i^{th}$ normal WSI, respectively. Moreover, let $H^{Normal}={\{H}_{1},H_{2},\ldots ,H_{N}\}$ denote the set of all normal-patch-level embeddings of all normal WSIs. Algorithm A1 (Appendix A) demonstrates how the NRK is constructed by means of a distinct feature vector identifier (DFI) given in Algorithm A2 (Appendix A). Figure 1 displays a schematic diagram of the NRK construction process. This process takes the normal WSIs as an input, utilizes the DFI module to select the distinct patch embeddings, and subsequently applies the DFI module on the aggregated distinct feature embeddings to select an optimal set of normal representative embeddings.

### 3.5. Instance Retrieval for WSIs Using Normal Representative Bag

In this section, we discuss how to employ the NRK obtained in Algorithm A1 to assign zero attention to certain normal patches, which are patches whose feature embeddings are lying in the negative (normal) subspace far from the positive (tumor) subspace. Note that at both training and inference times, the NRK singles out certain normal patches for both normal and tumor WSIs. Given the set of patch-level embeddings, $H_{q}=${$h_{q1},h_{q2},.h_{qn_{q}}$}, of a WSI, namely, $X_{q}$, we first construct the similarity matrix S∈ $R^{n_{q}\times m}$, where $m=cardinality\left(NRK\right)\;and$ the entry in the $i^{th}$ row and $j^{th}$ column of S is

(5) $$s_{ij}=\frac{h_{qi}^{T}k_{j}}{\left||h_{qi}||||k_{j}|\right|},$$

for $i=1,\ldots ,n_{q}$, and j=1,…,m. Note that the $i^{th}$ row of the similarity matrix S is a vector whose entries are the cosine similarity scores between $h_{qi}$ and NRK keys. To identify certain normal patch-level embeddings, which are embeddings corresponding to certain normal patches, we assign a normality score to each $h_{qi}$, for $i=1,\ldots ,n_{q}$, by

(6) $${⍺}_{i}=Avg\left(TopK\left(S_{i}\right)\right)$$

where $S_{i}$ is the $i^{th}$ row of the similarity matrix S, $TopK\left(.\right)$ is an operator that returns the top K values of an input vector, and $Avg\left(.\right)$ is the averaging operator. We then sort $H_{q}=${$h_{q1},h_{q2},.h_{qn_{q}}$} based on their normality scores, ${⍺}_{q1},{⍺}_{q2},.{⍺}_{qn_{q}}$ in descending order, and construct an ordered set, namely, $H_{q}^{Sorted}$. We finally select the bottom *r* percentile of $H_{q}^{Sorted}$ as uncertain patch-level embeddings, which are embeddings that can correspond to a tumor or normal patches within the WSI $X_{q}$, and are denoted by $H_{q}^{Uncertain}$. Note that we consider top-(100-*r*) percentile of $H_{q}^{Sorted}$ as certain normal patch-level embeddings within WSI $X_{q}$, and denoted by $H_{q}^{Certain}$. Figure 2 demonstrates how bottom *r* percentile embeddings (uncertain patch-level embeddings) of a WSI are selected and fed into the ABMIL model for a WSI-level label prediction.

### 3.6. Implementation Details

To extract the tissue region from the WSI, we apply the color thresholding method to extract the foreground tissue patches and discard the patches with more than 25% of the background region. Then, we crop the tissue region into 224 × 224 non-overlapping patches under 20× magnification. We used the ResNet50 model [25] (truncated after the third residual block) pretrained on the ImageNet dataset [26] that generates 1024-dimensional patch embeddings, and used CTranspath [27] as the histopathology pretrained feature encoder that generates 768-dimensional feature embeddings from the foreground tissue patches. We employed the aforementioned encoders separately to assess the effectiveness of the proposed method. During the training process, we used Adam Optimizer [28], 0.0002 learning rate, 0.00001 as weight decay, and 1.20:1 as the rescaling weight for tumor, and normal class. We use the early stopping strategy with a patience of 10 epochs after 30 warmup epochs. For the Camelyon16 experiment, we performed fivefold cross-validation with a 90:10% random split in the training set in each fold. Then, we evaluated our method on the official testing set of Camelyon16. The proposed method consists of three hyperparameters with the following range of values: σ(0.92–0.96), r(0.10, 0.20, 0.30, 0.50), and K(1, 5, 10, 20, 50, 100, 150). Here, K represents the Top-K similarity scores of each patch embedding with the NRK, and r represents the percentage of patches that are most dissimilar to NRK. We tuned these parameters based on the validation AUC and reported the results with K = 5, r = 0.10 (10% of the WSI patches), σ=0.95. Furthermore, we used the AUC, accuracy, recall, precision, and F1 score as the evaluation metrics for WSI classification.

For the experimentation involving the combined Camelyon16 and Camelyon17 [21] datasets, we divided the training data from Camelyon17 centers and Camelyon16 into an 80–20% ratio. We further divided the training set into 90% for model training and 10% for model validation. Subsequently, we generated keys from the newly created training data of each center using a value of σ=0.90. These keys were then combined, and a lower value of σ=0.80 was used to select a reduced number of keys that met the computation requirements. We used the same value of K,r ensuring consistency in the experimental setup. For training the model, we used the early stopping strategy with a patience of 20 epochs after 5 epochs.

## 4. Results

In this section, we evaluate the experimental results of the proposed method with the state-of-the-art methods and conduct an ablation study, and interpretability of the patch-selection method using NRK.

### 4.1. Results on WSI Classification

**Figure 3:**
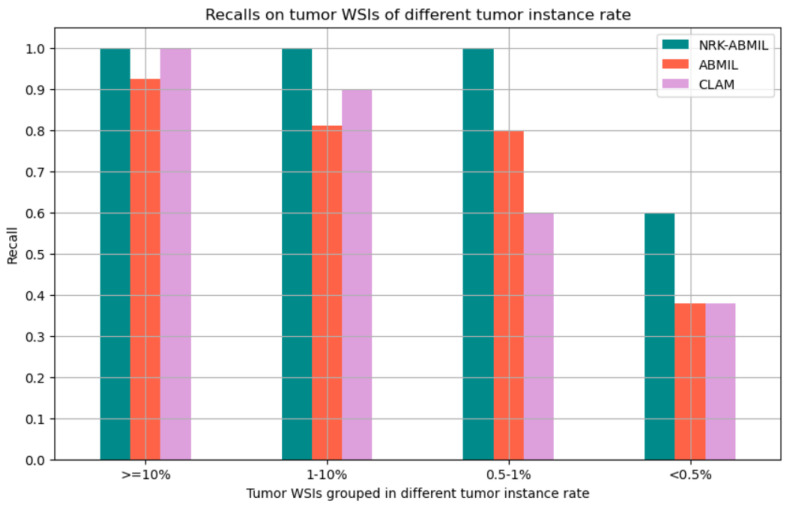
Recall of tumor WSIs based on tumor grouped by tumor instance rate.

We evaluated the effectiveness of the proposed method by comparing it to existing deep learning methods [8,9,10] on the Camelyon16 and Camelyon17 datasets. The results for [8,9,10] were computed using their official implementation. Specifically, for DSMIL [14], we retrained the feature extractor on the official training set of Camelyon16 [29]. Table 1 presents the results obtained using the ResNet50 feature extractor [25] on the Camelyon16 dataset. The proposed method outperformed the others, with an average AUC of 0.8967, and we observed an increase of 8.4% in AUC compared to the baseline (ABMIL) that applies attention to every instance of the WSI (Table 1). For the remainder of experimental evaluation, we conducted a comparative analysis between the proposed method and the most effective existing methods selected from Table 1 [8,9]. Table 2 presents the results obtained using the CTranspath feature extractor [27] on the Camelyon16 dataset. The proposed method achieved an average AUC of 0.9540 using the CTranspath feature extractor. Since the feature extractor trained on histopathology data surpasses the ResNet50 feature extractor [25] on the Camelyon16 dataset, we utilized the histopathology trained feature extractor to assess the performance of the proposed method on the Camelyon16+Camelyon17 dataset. Correspondingly, we observed an average AUC of 0.9334 on the Camelyon16+Camelyon17 dataset, and the detailed results are presented in Table 3. To evaluate the significance in terms of small-lesion detection on the Camelyon16 dataset, we assessed the efficiency of the proposed method by categorizing the lesions according to their size. We grouped the positive WSIs into four groups: (i) <0.5% (slides where the tumor is less than 0.5% of the tissue area), (ii) 0.5–1.0%, (iii) 1–10%, and (iv) >=10%. Figure 3 presents the comparison of the MIL models that use the ABMIL as the baseline. These findings unequivocally indicate that the proposed method exhibits sensitivity to small lesions without compromising its effectiveness in detecting large lesions.

### 4.2. Ablation Studies

**Figure 4:**
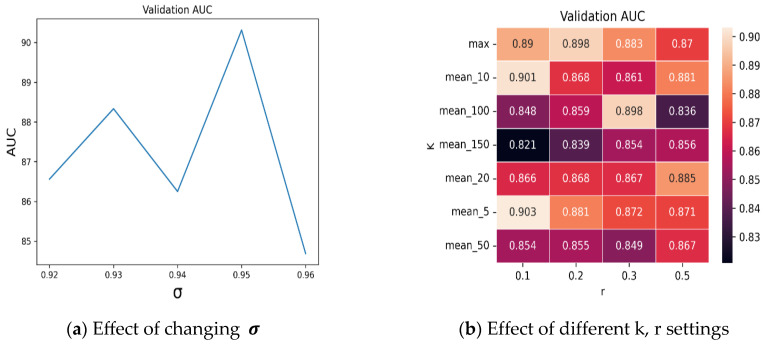
Ablation study of hyperparameters. (**a**) Effect of changing σ for NRK generation. (**b**) Effect of changing k, and r on the validation set. All of these metrics are the averaged fivefold validation AUC.

The goal of an ablation study is to investigate the impact of individual hyperparameters on the performance of a model, helping to determine their relative importance and optimize their values using a validation set. We conducted an ablation study to validate the effectiveness of key hyperparameters: K, r, and σ. To validate the impact of σ, we generated multiple NRK bags by setting σ=0.92, 0.93, 0.94, 0.95, and 0.96. We then evaluated the average validation performance of our method on each NRK bag. From Figure 4a, it can be observed that we achieved the best validation performance when σ=0.95 was used. Similarly, we present the mean validation AUCs of different k and r settings. As shown in Figure 4b, we achieved the best performance when the (k = 5, r = 0.10) pair was used.

### 4.3. Visualization and Interpretability of NRK-ABMIL

**Figure 5:**
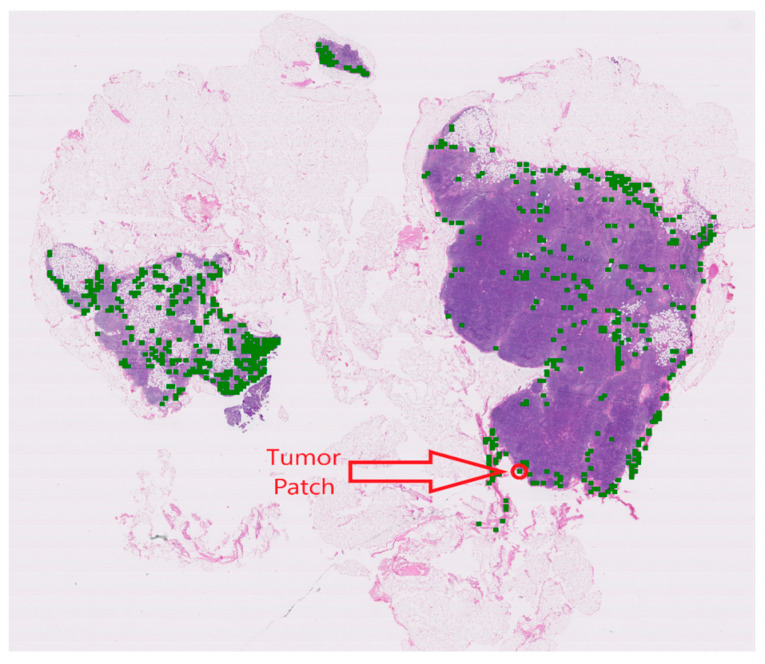
Visualization of selected patches from a tumor WSI. An example of tumor WSI. Selected patches from a tumor WSI are overlaid on top of WSI using green.

**Figure 6:**
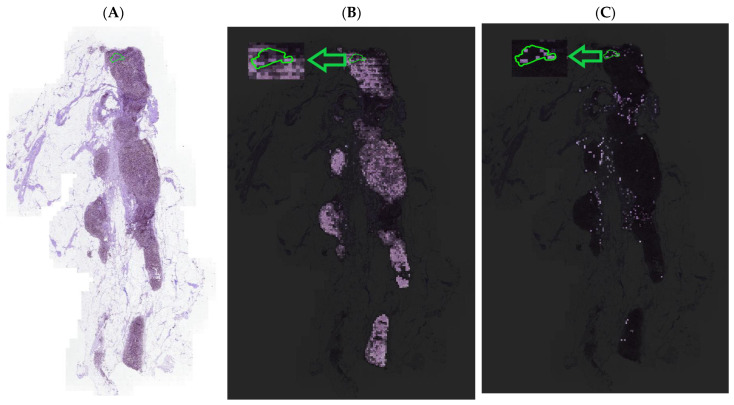
Comparison of attention maps between the current and NRK-ABMIL. Heatmaps are generated by mapping the attention weights to the corresponding regions of the WSI. (**A**) An example of tumor WSI showing a small lesion. (**B**) Attention map of tumor WSI using ABMIL. (**C**) Attention map of tumor WSI using NRK-ABMIL.

The importance of removing the normal patches is depicted in Figure 5. It presents a tumor WSI from the Camelyon16 dataset. Here, a red circle annotates the presence of a tumor lesion in the WSI. Green patches show the selection of the lowest similarity score patches with the NRK. From Figure 5, it can be seen that the proposed method is capable of selecting the small lesions and selecting the patches from the different regions of the WSI. Figure 6 shows the comparison of attention maps between ABMIL and NRK-ABMIL, revealing that NRK-ABMIL generates more precise attention maps than ABMIL.

## 5. Discussion

In this article, we introduce NRK-ABMIL, a weakly supervised learning model designed for tumor WSI classification. The proposed method uses a novel discriminative normal representation learning approach that identifies the discriminative normal representations from each WSI using a DFI module and generates a normal representation keyset (NRK). We then compare the NRK with WSI feature vectors for the selection of potential tumor patches within the WSIs. The identified patch embeddings are then fed into the MIL model for slide-level classification, enhancing the classification performance.

The proposed model achieved an average AUC of 0.8967 and 0.9540 using ResNet50 Feature Extractor [15] and histopathology-specific feature extractor [27] on the Camelyon16 dataset. Similarly, we achieved an average AUC of 0.9334 on Camelyon16+Camelyon17 for BCLNM classification, which surpasses the current state-of-the-art MIL models. In addition, our experimental results reveal that NRK-ABMIL outperforms other methods in terms of recall, particularly on microlesion tumor WSIs (see Table 1 and Figure 3). To ascertain the validity of the proposed method, we conducted an evaluation by merging the Camelyon16 and Camelyon17 datasets, and the results presented in Table 1, Table 2 and Table 3 highlight the potential of the proposed method in detecting metastasis. These findings suggest that selecting potential tumor patches for the MIL model is crucial for tumor WSI classification. As illustrated in Figure 5, the patch-selection module employed in NRK-ABMIL selects tumor patches from small tumor lesion areas, which proves the interpretability of NRK-ABMIL’s results. The attention maps shown in Figure 6 show that the proposed model focuses more on identifying areas with tumors, even on small lesions, and pays more attention when making its predictions. In this case, the models assign more weight to areas with tumors, which potentially improves the ability to detect small lesions. In comparison to the previous instance selection-based MIL method, the proposed NRK-ABMIL achieved better overall performance, especially in terms of recall on microlesion tumor WSIs. The improved performance can be attributed to NRK’s ability to learn a less redundant normal representative keyset, resulting in more robust instance selection.

A limitation of the proposed method is that our NRK module and the subsequent instance selection module rely on feature embeddings generated by a fixed ResNet encoder or pretrained CTranspath encoder without fine tuning on a target dataset, which can result in selection of patches that might not be separable in this feature space. Therefore, while our current method provides excellent performance for the driving problem we studied in this paper, there is room for improvement through the exploration of self-supervised learning models [30,31]. Another possible limitation of the proposed method is its sensitivity to tissue-stain inconsistencies. To overcome this issue, it is important to ensure that the keyset contains the representative keys for different data sources.

Despite the limitations, the proposed NRK-ABMIL provides a powerful automatic solution for tumor WSI classification. The proposed method can not only provide accurate slide-level prediction but also generate sparser and more tumor-oriented attention maps than other MIL methods.

The clinical significance of this method lies in its potential to help oncologists accurately identify breast cancer metastasis to lymph nodes, which is crucial for determining the stage of breast cancer. This method can be utilized in the development of improved treatment plans, as the detection of lymph node metastasis of small lesions is critical for improving the prognosis. An interesting application of the proposed method could be for the detection in the frozen section slides. These frozen slides often pose challenges in recognizing such small metastatic deposits, making their detection difficult. False-negative cases in frozen tissue can have serious consequences for patients and complicate care planning. This method can also lighten the burden on pathologists by offering highly precise ROI suggestions in areas where there is a shortage of skilled pathologists.

## 6. Conclusions

In this study, we propose a novel approach for classifying whole slide images (WSIs) with small lesions in a more precise and accurate manner. Specifically, we introduced a distinct feature vector identifier module as part of our normal representative keyset-based MIL approach, which allows for the selection of patches that are most relevant for accurately classifying WSIs. To evaluate the effectiveness of the proposed method, we conducted comprehensive experiments on the Camelyon16 and Camelyon17 datasets, which are widely used as benchmark datasets for evaluating computer-aided diagnosis systems for breast cancer metastasis. Our results demonstrated that the proposed NRK-ABMIL approach with the DFI module achieved excellent performance for accurately identifying small tumor regions within WSIs. The proposed method needs to be refined and validated for multiclass classification problems and using other medical use cases. We expect that the proposed method will generalize well, especially in accurately detecting small lesions within WSIs. In our future studies, we plan to test our proposed method for other types of cancer.

## Figures and Tables

**Table 1:** Testing results on Camelyon16 dataset using ResNet50 Feature Extractor [25]. In each entry of the table, we report averaged testing results with standard deviation (top row) and testing results achieved by the best validation model (bottom row) across five folds (best evaluation metrics are highlighted in bold).

Method	AUC	Precision	Recall	F1
ABMIL [10]	0.8127 ± 0.034 0.8375	0.9108 ± 0.0759 0.8684	0.6327 ± 0.0827 0.6734	0.7392 ± 0.040 0.7586
CLAM [8]	0.8580 ± 0.027 0.8319	**0.9120** ± **0.009** 0.8462	0.6780 ± 0.024 0.6735	0.7770 ± 0.016 0.7500
TransMIL [9]	0.8500 ± 0.028 0.8403	0.8312 ± 0.030 0.8471	0.7898 ± 0.041 **0.7913**	0.7990 ± 0.040 0.8182
DSMIL [14]	0.8294 ± 0.036 0.8277	0.9077 ± 0.052 **0.9285**	0.6485 ± 0.036 0.6533	0.7590 ± 0.032 0.7669
Ours	**0.8967** ± **0.016** **0.9007**	0.8589 ± 0.044 0.8837	**0.8000** ± **0.041** 0.7755	**0.8269** ± **0.0265** **0.8239**

**Table 2:** Testing results on Camelyon16 dataset using CTranspath Feature Extractor [27]. In each entry of the table, we report averaged testing results with standard deviation (top row) and testing results achieved by the best validation model (bottom row) across five folds (best evaluation metrics are highlighted in bold).

Method	AUC	Precision	Recall	F1
CLAM [8]	0.9339 ± 0.015 0.9533	0.8913 ± 0.062 **0.9756**	0.8489± 0.033 0.8163	0.8673 ± 0.019 **0.8888**
TransMIL [9]	0.9394 ± 0.009 0.9313	**0.9054** ± **0.062** 0.8723	0.8286 ± 0.042 **0.8367**	0.8623 ± 0.013 0.8541
Ours	**0.9540** ± **0.015** **0.9701**	0.8997 ± 0.047 0.9750	**0.8489** ± **0.030** 0.7959	**0.8723** ± **0.019** 0.8764

**Table 3:** Testing results on Camelyon16+17 dataset using CTranspath Feature Extractor [27]. In each entry of the table, we report averaged testing results with standard deviation (top row) and testing results achieved by the best validation model (bottom row) across five folds (best evaluation metrics are highlighted in bold).

Method	AUC	Precision	Recall	F1
CLAM [8]	0.9305 ± 0.015 0.9208	0.8404 ± 0.066 0.8996	0.8101± 0.042 0.8290	0.8219 ± 0.022 0.8628
TransMIL [9]	0.9221 ± 0.012 0.9180	0.8544 ± 0.028 0.8752	0.8250±0.035 0.8146	0.8389 ± 0.025 0.8438
Ours	**0.9334** ± **0.008** **0.9254**	**0.9083** ± **0.053** **0.9772**	**0.8372** ± **0.031** **0.8333**	**0.8694** ± **0.012** **0.8995**

## Data Availability

Camelyon16 slides are available from the ISBI challenge on cancer metastasis detection in lymph node (https://camelyon16.grand-challenge.org/Data/, accessed on 10 December 2021). Camelyon17 slides are available from the Grand Challenge website (https://camelyon17.grand-challenge.org/Home/, accessed on 4 April 2022). Code will be available at https://github.com/cialab/NRKMIL, accessed on 4 April 2022.

## Appendix A

**Algorithm A1** Normal representative keyset (NRK)
**Input:** The set of normal WSIs, $H^{\mathrm{Normal}}=\{H_{1},H_{2},\ldots ,H_{N}\}$, and a similarity threshold $\sigma \in \left(0,1\right)$.
**Step 1: for** i=1,2,…,N do
- Set $NRK_{i}=DFI(H_{i})$, where the module DFI is given in Algorithm A2.
**End (for)**
**Step 2:** Set $NRK\;=DFI(\underset{i=1}{\overset{N}{⋃}}NRK_{i})$= {$k_{1},k_{2},.,k_{m}$}.
**Output:** NRK = {$k_{1},k_{2},.,k_{m}$}.

**Algorithm A2** Distinct feature vector identifier (DFI)
**Input:** A set of feature vectors $H=\{h_{1},h_{2},\ldots ,h_{n_{H}}\}$ and a similarity threshold $\sigma \in \left(0,1\right)$, where $h_{j}\in R^{D\times 1}$ for $j=1,2,\ldots ,n_{H}$. Moreover, set DF={} (empty set).
**Step 1:** Compute $B=\{b_{1},\;b_{2},.b_{n_{F}}\}$, where $b_{j}=\frac{h_{j}}{∥h_{j}∥}\in R^{D\times 1}$, for $j=1,2,\ldots ,n_{H}$.
**Step 2: While** B≠{} (empty set) do
- **Step 2.1:** Set $b_{R}=popRand\left(B\right)$, where $popRand\left(.\right)$ is a function that randomly selects only one element of its input set, i.e., H.
- **Step 2.2:** Compute
$C=\left[\begin{array}{c} c_{1},\;c_{2},\ldots ,\;c_{\left(R-1\right)},\;c_{\left(R+1\right)},\ldots ,\;c_{n_{H}} \end{array}\right]\in R^{1\times \left(n_{H}-1\right)},$
where $c_{j}=b_{R}^{T}b_{j}$ for $j=1,2,\ldots ,R-1,R+1,\ldots ,n_{H}.$
- **Step 2.3: for** $j=1,2,\ldots ,R-1,R+1,\ldots ,n_{H},$ do
If ($c_{j}$ ≥σ), then
$B=B.\;remove\left(b_{j}\right)$,
where $A.\;remove\left(a\right)$ is a function that removes element a from set A.
**End (for)**.
- **Step 2.4:** Set
$DF\;=\;DF\;\cup \;h_{R},$
And
$B=B.\;remove\left(b_{R}\right).$
**End (While)**
**Output:** DF
